# The Future of Digital Sequence Information for Plant Genetic Resources for Food and Agriculture

**DOI:** 10.3389/fpls.2019.01046

**Published:** 2019-08-30

**Authors:** Sylvain Aubry

**Affiliations:** ^1^Department of Plant and Microbial Science, University of Zurich, Zurich, Switzerland; ^2^Section Genetic Resources and Technology, Swiss Federal Office for Agriculture, Bern, Switzerland

**Keywords:** plant genetic resources, digital sequence information, International Treaty for Plant Genetic Resources for Food and Agriculture of the Food and Agriculture Organisation, digitization, plant genetic resources for food and agriculture

## Abstract

The recent debates on the legal status of “digital sequence information” (DSI) at the international level could have extensive consequences for the future of agriculture and food security. A large majority of recent advances in biology, medicine, or agriculture were achieved by sharing and mining of freely accessible sequencing data. It is most probably because of the tremendous success of modern genomics and advances of synthetic biology that concerns were raised about possible fair and equitable ways of sharing data. The DSI concept is relatively new, and all concerned parties agreed upon the need for a clear definition. For example, the extent to which DSI understanding is limited only to genetic sequence data has to be clarified. In this paper, I focus on a subset of DSI essential to humankind: the DSI originating from plant genetic resources for food and agriculture (PGRFA). Two international agreements shape the conservation and use of plant genetic resources: the Convention on Biodiversity and the International Treaty for Plant Genetic Resources for Food and Agriculture. In an attempt to mobilize DSI users and producers involved in research, breeding, and conservation, I describe here how the increasing amount of genomic data, information, and studies interact with the existing legal framework at the global level. Using possible scenarios, I will emphasize the complexity of the issues surrounding DSI for PGRFA and propose potential ways forward for developing an inclusive governance and fair use of these genetic resources.

## Introduction

In his Discourse of Inequality, Rousseau states: “You’re lost, if you forget that the fruits of Earth belong equally to all of us, and Earth itself to nobody” (Rousseau, 1755). Similar thoughts were surely some of the underlying motivations of negotiators during the development of the International Treaty on Plant Genetic Resources for Food and Agriculture (PGRFA) in the early 2000, later referred here as the “Seed Treaty” (Esquinas-Alcazar et al., 2013; ITPGRFA, 2004). Since more than a century, the concept of genetic resources frames breeding and conservation into a genocentric perspective that is intertwined with their own erosion and the danger of losing biodiversity (Bonneuil, 2019; Harlan, 1975). As “fruits of the Earth” are becoming scarce in quantity and quality (Esquinas-Alcázar, 2005; Khoury et al., 2014), there is more than ever a need for fair and equitable governance of genetic resources, especially those relevant for breeding new crops and food security (Genetic Resources for Food and Agriculture, GRFA).

**Figure 1:**
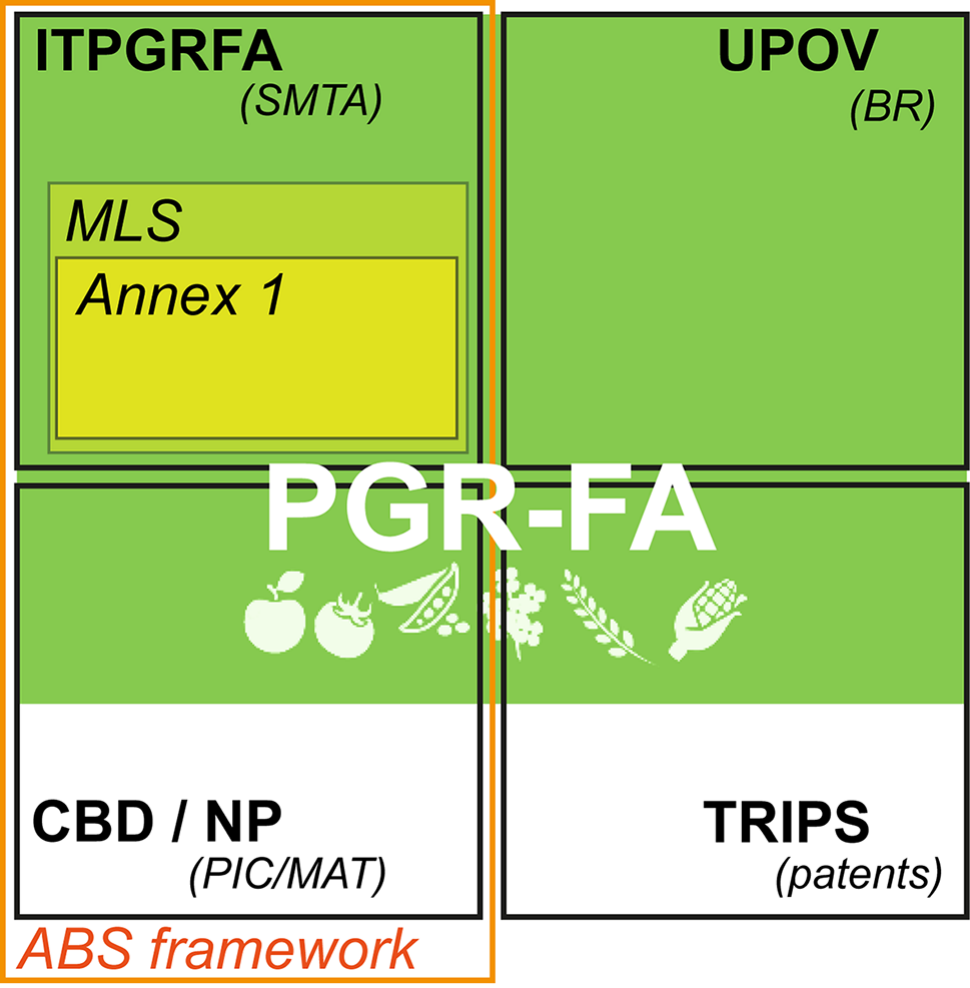
The regime complex regulating Plant Genetic Resources for Agriculture (PGRFA). ITPGRFA, International Treaty for Genetic Resources for Food and Agriculture; SMTA, Standard Material Agreement; MLS, Multi-lateral System; UPOV, International Union for the Protection of New Varieties of Plants; BR, Breeder's right; CBD, Convention for Biodiversity; NP Nagoya Protocol; PIC/MAT, Prior Informed Consent/Mutually Agreed Terms; TRIPS, Agreement on Trade-Related Aspects of Intellectual Property Rights; ABS Access and Benefit Sharing.

Two international agreements shape the conservation and use of PGRFA: the Convention of Biodiversity (CBD) that rules any terrestrial genetic resources and the Seed Treaty, which apply only on a subset of species relevant to agriculture and food security. Both are the main elements of the access and benefit sharing framework (**Figure 1**). The main objectives of ABS framework are summed up in the Article 2 of the CBD: to insure “conservation of biological diversity, the sustainable use of its components and the equitable sharing of benefits arising from the utilization of genetic resources” (CBD, 1992). The Seed Treaty has been designed as a sector-specific reply to the CBD (Esquinas-Alcazar et al., 2013; Manzella, 2013) taking into consideration the need of a global commons to counteract loss of agricultural biodiversity and insure food security. It was also perceived as a global effort to counteract the trend in privatization of PGRFA, including the challenge associated with recognition of the State sovereignty over genetic resources highlighted by the CBD (Halewood et al, 2013). The Seed Treaty remains one of the most remarkable attempts to create a global commons around phytogenetic resources that recognize farmers as essential for their conservation and sustainable use (FAO, 2010; Halewood, 2013). It also aims at facilitating access to plant genetic resources for farmers, conservationists, breeders, scientists, and teachers. It is a result of a multi-decade process of international negotiations that constrained its design (Halewood et al., 2013). For example, the boundaries of the common are strictly limited. The core of the Seed Treaty’s common, the multilateral system, comprises a pool covering 64 of some of the most important species for agriculture. These 64 species, listed in the annex 1 of the treaty, contribute to an estimated 90% of calories, fat, protein, and weight consumed worldwide (ITPGRFA, 2004; Khoury et al., 2014). The multilateral system is often referred to as an example of global management system of the global common pool of resources (Halewood et al., 2013). Noteworthy, as a result of difficult and protracted negotiations, it only represents a fraction of the entire PGRFA (Manzella, 2013; Khoury et al., 2014). In fact, species listed in annex 1 are only part for the multilateral system when exclusively used for food or feed purposes and when those resources are under the “national governments management and control systems” (ITPGRFA, 2004). Some voluntary inclusions to the multilateral system are also possible. In addition, the multilateral system also excludes material that is declared “under development.” Some important key agricultural species are not in the annex 1 (like coffee, soybean, or sugarcane), leading to some future discussion about extension or suppression of the annex 1.

Any PGRFA falling out of the scope of the Seed Treaty and not ruled by any other intellectual property regime would then eventually be regulated by the CBD and its Nagoya Protocol. Under the Nagoya protocol, the sovereignty of the country of origin is prevalent, and each single resource exchange negotiated on a case-by-case basis. All species within the multilateral system are governed by a standard material transfer agreement (SMTA) whereas the Nagoya Protocol regulates access to the resources based on a system of bilateral agreements between countries, using prior informed consent and mutually agreed terms (PIC/MAT). The SMTA used in the Seed Treaty is a standardized contract that allows a facilitated exchange of resources between signatories. Also, being the result of complex negotiations (Esquinas-Alcazar, 2013), it allows coordination between the global/international level and local stakeholders. One of its specificity is to set a default rate of benefits to be given to a third party, namely, a fiduciary fund managed by the FAO.

In a similar way to the CBD, the Seed Treaty by definition focuses on physical genetic resources: “any material of plant origin, including reproductive and vegetative propagating material, containing functional units of heredity” (ITPGRFA, 2004). However, it remains unclear to which extent the benefit sharing obligations included in the SMTA of the multilateral system do also concern obligations toward digitally derived data (often referred to as “digital sequence Information,” DSI) (CBD/COP/DEC/XIII/16, 2016). Considering genetic resources exclusively as a material entity (mostly germplasm, seeds, seedlings…) may misjudge the modern practices related to GRFA and the exact nature of what is being “extracted” from these. This potentially precludes these instruments to adapt to today’s modalities of use of the genetic resources: entities that are primarily mined to generate large amounts of (digital) data resulting from the use of various “omics” approaches. Previous to the digitization of genetic resources, the material-centric definition of PGRFA has already been criticized by some scholars proposing an alternative governance framework of PGRFA, which would have valued natural information (Vogel, 2011; Ruiz Muller, 2015).

A complex set of partially overlapping international legally binding instruments frame intellectual properties of PGRFA that have distinct underlying principles and aims (**Figure 1**, Roa-Rodríguez and van Dooren, 2008). The main relevant instruments for PGRFA are trade secret protection, patents, breeder’s rights, copyright, and sovereign right over generic resources (for a comprehensive review, see Winter, 2013). Either one or both physical (seeds) and informational (genomic sequences) entities could in principle be privatized under those regimes (Winter, 2013; Roa et al., 2016). As a consequence, a single PGRFA could in theory be privatized based on a whole range of criteria/justifications (inventivity, genetic “potential,” country of origin…) depending whether their informational or physical (or both) aspects are being considered and which regime is considered. There is a large body of work commenting on advantages and limits of each of these instruments, especially on the extent to which the global trend to privatize genetic resources impact on the PGRFA common (Dedeurwaerdere, 2013; Winter, 2013). It appears therefore that negating the hybrid status of PGRFA: both a physical and informational commodity makes it hard to design a policy framework that could fit in this “regime complex” (Raustiala and Victor, 2004) in order to guarantee access and sharing of the potential benefits. At first, an appropriate PGRFA definition seems therefore necessary to adapt to the actual state of scientific progress.

Since 2016, debates about the status of DSI has gained prominence in several international circles, including the ABS framework: the Seed Treaty (ITPGRFA, 2015; Manzella, 2016), the CBD, but also the Convention on International Trade of Endangered Species, the Pandemic Influenza Preparedness Framework of the World Health Organization, and the United Nations Convention on the Law of the Sea. This triggered a great deal of confusion and a subsequent surge of analyses and reports (CBD/COP/DEC/XIII/16, 2016; Marden, 2017; Welch et al., 2017; Heinemann et al., 2018; Laird and Wynberg, 2018). The development of synthetic biology, in other words, our increasing abilities to read and write DNA (Chari and Church, 2017), has cast fear on the possibilities to overrun physical access. However, it is possible that the DSI issue only brought to light a pre-existing ontological weakness (or unresolved long-standing tension) of the genetic resources definition in international agreements (Deplazes-Zemp et al., 2018; Marden, 2018; Prathapan et al., 2018). Here, I try to explain why a merely technical evolution that allowed a more exhaustive description of the PGRFA, namely, high-throughput sequencing of genetic information (and other “omics” data), has led to challenge the very basic fundaments of PGRFA governance and describe some possible ways forward.

## Digital Sequence Information: Another Face of “Big Data”

Until recently, the terminology “DSI” was mostly unknown to a majority of scientists (Heinemann et al., 2018). In addition, it still remains largely unclear whether DSI should be only restricted to nucleotide sequences (“genomic information”) or should also integrate other omics data like other nucleic acids, methylation status, metabolites, or even phenotypic data (Halewood et al., 2018a). Independently of the unavailability of any internationally agreed definition, genomics alone generates petabases (10^15^) of data (i.e., nucleotide sequences) every year (www.ncbi.nlm.nih.gov/sra) and is predicted to soon exceed other “big data” science in size and complexity (Stephens et al., 2015; Heinemann et al., 2018). The quick rate of DSI generation emphasizes the potential need for a global standardized infrastructure to ensure long-term data preservation (Leonelli et al., 2013). Independently of the speed of knowledge and data generation, the “digitality” of genomic data seems trivial in practice, and DSI could well be considered as presenting most of the attributes of other “digital artifact” produced in other domains, where alternative governance models have already been implemented successfully (Kallinikos et al., 2013). Research on PGRFA also leads many initiatives to open possibilities of DSI management globally, like DivSeek for crop genomics data (www.divseek.org) or GODAN for phenotypic data (www.godan.info). Similarly, conservation of PGRFA tends to include increasing amounts of DSI, like in the DNA barcoding of life initiative (http://www.boldsystems.org/), “local” initiatives like the sequencing of genomes of an entire botanical garden (Liu et al., 2019), or major efforts from the Consortium of International Agricultural Research Centers (CGIAR) to “digitally curate” their collections (Halewood et al., 2018b). Ambitious projects of large-scale genome sequencing, like the “Earth BioGenome project” planning to sequence not less than 10 million eukaryotic species, will also bring new insights likely to be relevant for food and agriculture (Lewin et al., 2018).

The prominence of big “omics” data in biological science has not only challenged the way science is done and shared but also its underpinning philosophy (Leonelli, 2014). At odds with the increasing complexity of the instruments ruling on intellectual property over the last 30 years, biology has gradually favored opening access to the public research data and results (Strasser, 2011; Sullivan, 2004). This has recently crystallized in various policy guidelines worldwide (Wilkinson et al., 2016) that are also widely advocated in synthetic biology [OpenPlant (BioBricks Foundation), 2015]. In this context, promoting open data is essential, but the “digital divide” inherent to modern use of information and communication technologies still must be considered (Bastow and Leonelli, 2010; Bezuidenhout et al., 2017). Inequalities in data access, infrastructures and experts should not stretch the already relatively libertarian design of the Seed Treaty (Thomas, 2014). Bezuidenhout and colleagues suggest that inequalities amongst various stakeholders (farmers, breeders, researchers, consumers) should be better integrated when it comes to build an effectively open data framework, in a very similar way to Sen’s approach to capability of human wellbeing (Sen, 1999). Open science (and open data) has been critically considered as shared between various owners that have a common monopoly on investments and on use of the openly accessible knowledge (Callon, 1994; Stengel et al., 2009). In other words, “open science” does not necessarily mean “fair science,” and “access to” can differ greatly from “utility of” DSI (Bezuidenhout et al., 2017).

A similar debate exists in synthetic biology about digital-only information originating from genomics datasets. Alternative IP models emerged to regulate exchange of DNA “parts” [OpenPlant (BioBricks Foundation), 2015; Welch et al., 2017]. There, a two-tier model distinguishing non-commercial technology from high-potential output was designed to maximize sharing of biomaterials and associated data. This approach was designed to answer the needs of this very specific research community (SynBio), and more work is needed to show to which extent that it might be transferable to PGRFA and breeding context at large.

The ambitious goal of the Seed Treaty is to allow “conservation and sustainable use of PGRFA, the fair and equitable sharing of the benefits arising from their use (…) for sustainable agriculture and food security” (Art. 1, ITPGRFA, 2004). These goals can be achieved, however, only with a more comprehensive and a better understanding of the way digitization has changed practices. When designing PGRFA governance, care should be taken to provide not only clear modalities of access and exchange of data, but also to the capabilities of each stakeholder involved. This would in turn allow an actual share of benefits (being monetary or not) from the global biodiversity for food and agriculture.

## Specificities of DSI-PGRFA Use in Plant Breeding

Plant research and crop improvement are largely dependent on access to DSI, being genotypic or phenotypic data (Spindel and McCouch, 2016; Halewood et al., 2018a). The Seed Treaty recognizes that exchange of information is a “non-monetary” benefit. However, given that the treaty’s design largely preceded most of the genomics’ developments, it remains unclear, to which extent this information exchange is considered in daily practice (Welch et al., 2017; Marden, 2018). Interestingly, some unique facets of the PGRFA are often overlooked when it comes to using DSI for modern breeding. PGRFA is a multifaced concept, involving many actors and being attached to a vast diversity of socio-economical values, and could be considered as a unique type of cultural commons (Ostrom and Hess, 2007; Madison et al., 2010; Halewood, 2013). Indeed, usage of PGRFA in a breeding program does not usually exhaust the resource (non-rivalry), but rather improves its intrinsic value and can even renew interest for its conservation. In addition, DSI originating from PGRFA will be open to all (non-exclusionary), even though this largely depends on who is able to perform the actual sequencing/phenotyping or which intellectual property regime may apply (Winter, 2013). Therefore, DSI originating from PGRFA need a tailored “new commons” concept to be adjusted to their particular hybrid status, and an extension of the ITPGRFA-framework seems the most pragmatic way to allow such a change of paradigm. DSI from PGRFA differs from the classic views on natural resources or cultural commodities by at least two main aspects:

- An optimal breeding value will be achieved, for example, during genomic selection, by merging pre-breeding data of multiple accessions. There is a necessary mixing and computing of large amounts of diverse accessions and lines in order to provide the targeted genetic progress or improved breeding values (Spindel and McCouch, 2016). This, in turn, will make the exact contribution of every single used “accession” hardly tractable. Indeed, one of the triggers of the onset of the multilateral system was the widely recognized spread of most of the plants used for food and feed worldwide (ITPGRFA, 2004). DSI add one degree of complexity to this pre-existing issue.
- The raise of synthetic biology, or more precisely the foreseen capacities of *de novo* synthesizing very large DNA fragments, even though not extensively used in breeding so far (Heinemann et al., 2018; Aubry and Eigenmann, 2019) disrupted the link between the material (germplasm) and its derived products. Despite being a very efficient system for describing and protecting DNA sequences for patents, it remains unclear how diverse and how pervasive any given intellectual property system should apply to DSI from PGRFA. For example, how easily could a codon-optimized resistance gene originating from a cryptogamic mushroom that is transformed into a modern variety of wheat be traced back to the organism it was first identified? Could intellectual property systems protect complex multi-gene networks that are essential for crop stress or pathogen resistance (Hickman et al., 2017)? How relevant is the protection or retention of data from crop pathogens that are constantly evolving and often represent global threats on agriculture (Sánchez-Vallet et al., 2018)?

The existing policy framework accommodates badly with the non-static, widely spread, non-rivalrous, and often non-exclusionary characters of DSI from PGRFA. There are fundamental differences between resources (to be extracted), natural genetic resources (to be extracted and valued), and PGRFA (to be primarily mixed/crossed to increase variability and selected in the process of breeding). These specificities need to be acknowledged when trying to improve coherence over the global governance of DSI-PGRFA.

Characterization/sequencing of PGRFA is regularly performed on cultivars, landraces, farmer’s breeds, or even crop wild relatives. This aims at linking phenotype and genotype and generally produces large amounts of (digital) data. Ultimately, the goal is to enable prediction of phenotypes based on genome-wide variability. This technique is referred to as “genomic selection” (Hamblin et al., 2011; Spindel and McCouch, 2016). Pan-genomes, genomes, transcriptomes, metabolomics, and phenotypic data can be used in genome-wide association studies (GWAS) to identify relevant traits, being a single allelic variant that is linked to an agronomic trait or to gene networks (Lipka et al., 2015; Halewood et al., 2018b). Genotyping-by-sequencing and GWAS frameworks were applied based on genome variability in a diverse array of crops like maize (Yano et al., 2016), sorghum (Morris et al., 2013), pearl millet (Varshney et al., 2017), chickpea (Basu et al., 2018), peanut (Zhang et al., 2017), banana (Sardos et al., 2016), cassava (Kayondo et al., 2018; Zhang et al., 2018), and cowpea (Burridge et al., 2017). Metabolite-based GWAS was also reported as an important tool to improve genomics-assisted selection for crop improvement (Fernie and Schauer, 2009; Luo, 2015). Increasing amounts of research programs are mining the genetic diversity already collected and readily available from gene banks. Successful attempts allowed to identify the genetic basis of traits responsible for fragrance in rice (Daygon et al., 2017), underlying genetics responsible for pearl millet drought resistance (Varshney et al., 2017), or loci encoding morphological diversity in barley (Milner et al., 2018). All of these genomic techniques allowed a better description (high-resolution fingerprinting) of the overall genetic diversity existing in each germplasm that, in turn, improve identification of relevant traits and allow a better breeding prediction and efficiency (Varshney et al., 2012). This ultimately helps ensuring a sustainable use of the PGRFA to provide crops that are locally adapted, resilient to various biotic and abiotic stresses, and necessary to maintain high levels of food security.

Noteworthy, identification and characterization of novel traits have been possible by merging several hundreds of accessions globally. The modalities of genomic data (DSI) use make traceability irrelevant: the value lies in the amounts of data analyzed, rarely in a single accession. However, exploiting the existing diversity collected in gene banks does not necessarily acknowledge the previous work of breeders and farmers during the course of agricultural history, and taking into consideration these resources as mere data-providing artifacts (“bulk of genes”) may also exclude central socio-economical relationship of farmers to their crops (Thomas, 2014; Bonneuil, 2019). Continuous exchange of genetic material has shaped a large majority of the breeding programs and follows self-established decentralized rules specific to every crop. However, it is unclear how the raise and diffusion of genomic data may integrate with these already existing structures, as well as their influence on the relationship between large and small breeders, and finally how an increasing amount of freely available data might influence practices. Several initiatives, like DivSeek (McCouch et al., 2013), have tried to provide a global accessible infrastructure to catalogue the world’s seed and derived genomic data collections.

## Assessment of Policy Options: Governing the Future of PGRFA

I have shown here actual modalities and challenges in considering some aspects of PGRFA as big data and try to evaluate the impact of digitization of PGRFA on the existing international policy framework. The various models described below extend on ongoing negotiations and by far are not meant to be exhaustive. I will evaluate *ex ante* their practicality and potential consequences (**Table 1**).

**Table 1:** Overview of possible options to include DSI-GRFA into existing genetic resources regulatory regime and their possible advantages and limits.

DSI governance models		Advantages	Limits	Challenges
*Exclusion*		Easy implementation	Do not reflect the actual state-of-the-art of the use of GRFA Risk of obsolescence of the treaties on GR globally	Will or have been already criticized by providers and mega-diverse countries as having possible large consequences on the ITPGRFA and CBD
*Extension of the SMTA to DSI*		Might make possible a better traceability and guarantee BS on DSI originating from specific GRFA	Practically almost impossible to set up Practicability will limit or impair overall use of GR(FA) and the rate of crop improvement.	Very hard to be globally implemented even though protection of DNA sequences well established for patent
Extending the common	*Subscription model*	Relatively easy within ITPGRFA. Might even be an incentive to extend the multilateral system	Limit already existing with the ITPGRFA. For example, limitation to the annex 1 crops and low or no participation to the Fund	Extend the scope of the treaties to any DSI involved in agriculture Reaching an international consensus
	*Bounded openness*	Simplify the access to GR(FA) Transparency on the benefits	Might hurt principles of sovereignty Large discretionary power of patent offices	Totally ignore potential socio-economical values of PGRFA and their associated DSI
	*Knowledge common*	Relative ease of use once running Allow an easy assessment of the global fairness of the system	Might be difficult to convince all stakeholder to take part	Would require a global effort to normalize multilateral governance on GRFA
*Common heritage of humankind*		Inherently open access	Have been shown in other fields not to be protecting fairness and taking into consideration capabilities of all stakeholders	Largely idealistic given the history of “common heritage” policy

This table provides a large panorama of the possible policies, from the easiest (exclusion of DSI) to the broadest (DSI as common heritage) models.

### Ignoring DSI: *De Facto* Open Access for Any Digital Information

Technically, the easiest solution is to ignore the DSI issue and to defend a *status quo*: keep the billions of sequenced DNA information away from the scope of any internationally binding treaty. A more holistic approach has been proposed, using the prominent situation of the CGIAR, to try inspiring new standards and soft norms to the field—for example, extension of the Global Information System (GLIS) infrastructure (Ker et al., 2013), or association of open-access passport data or digital object identifier (DOI) systematically to each shared element from a PGRFA (Manzella, 2016; Roa et al., 2016; Halewood et al., 2018a). For accessions not contained in the multilateral system, it has also been suggested to exclude non-commercial research from any data-access restriction (Biber-Klemm et al., 2010). Indeed, there are obvious contradictions when limiting access to data in conservation biology: this could possibly impair our understanding of the extent of biodiversity loss (also observed for cultivated plants and their relatives) and, therefore, limits our option to counteract it (Deplazes-Zemp et al., 2018; Prathapan et al., 2018; FAO, 2019).

As described in the previous section, sustainable use and conservation of PGRFA will require maximizing the amount of data available. For example, the necessary conservation of crop wild relatives (CWR) and their description are essential for expanding the gene pools used in modern crop breeding (Brozynska et al., 2016; Gruber, 2016; Capistrano-Gossmann et al., 2017; Dempewolf et al., 2017).

Ignoring the digitality of PGRFA might be the most pragmatic way of processing, in view of the quantity of already freely available data in public databases, but it also vastly ignores issues related to inequalities of access and capacities to value these data for a sustainable use in agriculture. Therefore, it remains to be evaluated what would be the exact consequences—for example, on global food security, of a purely libertarian stand on DSI-PGRFA. Similarly, consequences of any regulation, or uncertainty about potential forthcoming regulations, on innovation are hardly predictable. Given the actual stand of the discussion, this option is unlikely to satisfy all parties, unless maybe guaranteeing a global access to any DSI from (any) PGRFA, including data from germplasm not necessarily outside the multilateral system of the Seed Treaty crops.

### Extension of the Standard Material Agreements to DSI

As shown in the previous section, the specific modalities of DSI use in breeding make any restriction to the access to DSI particularly difficult and probably irrelevant: the potential value of a PGRFA results very often from its collective use and changes with time. In addition, issues related to the actual jurisdiction in which a PGRFA could originate, and how common any given trait, variant, or metabolite is to another PGRFA, make any possible tracing system elusive. For example, meta-studies of soil microorganisms would hardly trace back to actually described species, nor would a specific microbe strain be doubtlessly associated with any given country of origin. Despite these difficulties, some attempts to track DSI, or at least to improve DSI transparency, are under discussion at the database level (Scott and Berry, 2016) and on various policy fora such as the Intergovernmental Committee on Intellectual Property and Genetic Resources and the Traditional Knowledge and Folklore of the World Intellectual Property Organization (WIPO, 2018). Beyond the obvious political difficulties in amending a new SMTA that would satisfy all parties, practically, the underlying peculiarities of the biology and the genetics associated with DSI will make any enforcement impossible and potentially counterproductive. Alternatively, if DSI from PGRFA have to be “incorporated” as benefits as in the terms of Art 13.2.d of the Seed Treaty, downstream uses of these data would have to be understood and acknowledged to try keeping the initial intension of this instrument regarding their sustainable use. Concerning species outside the scope of the multilateral system, the issue regarding DSI is probably even more pronounced, and access to the data might only be a matter of voluntary contribution of the DSI producers to the public databases.

In any case, in view of the DSI specific properties (non-rivalry, enhanced value through their use and geographic “triviality”), it might become relevant in the next future to reconsider—at least concerning DSI—whether the access and the benefit sharing should remain coupled the way they are now.

### Extending the Commons

During the 6^th^ and 7^th^ sessions of the Governing Body of the Seed Treaty, an extension of the multilateral system to a subscription model has been discussed (ITPGRFA, 2015; ITPGRFA, 2017). Despite being part of another discussion about possible general improvement of the instrument itself, this discussion indirectly revealed the multilateral system of the Seed Treaty prone to fit with the complexity of DSI, especially compared with other ABS instruments (Halewood et al., 2018a). However, the exact modalities to possibly integrate DSI to such a system could greatly influence the way PGRFA would be further used/shared, the behaviors of various stakeholders, and finally the relevance of the existing instruments. In view of the complexity of the regulation of genetic resources, whatever the next genetic resource-related governance design would be, this will necessarily have influences on other fields. For example, it is unclear how much the rather restricted commons designed by the World Health Organization as especially dedicated to the governance of the influenza virus (and includes DSI) might serve as a model for other treaties (Fidler and Gostin, 2011). To summarize, three alternative multilateral models could be envisaged to cope with DSI (**Table 1**):

- The subscription model as a “simple” way to consider DSI. Ongoing discussion to extend the multilateral system could represent a solution to integrate the “informational” component of PGRFA into the scope of the Seed Treaty while keeping administrative burden low. A front payment would allow access to a multilateral system DSI database of PGRFA (like GLIS). Given there would be no “parallel system” running (to avoid free-riding), this model could allow more transparency and fairness. However, some parties already announced they would not participate to any subscription system if DSI were to be taken into consideration (ITPGRFA, 2017). Such adaptation of the multilateral system to the modern modalities of DSI use should be taken as a unique chance to make the treaties closer to modern practice. A subscription model might also be able to uncouple the actual DSI use and the probable commercialization of the derived information. In any case, to have a chance to be globally accepted and implemented, this will require a high level of trust from all stakeholders.
- An alternative multilateral system, the “bounded openness” has been advocated as a way to integrate DSI as natural “intangible” information (Ruiz Muller, 2015; Vogel et al., 2018). One of the basic fundamental point of this model is the new definition of genetic resources as “any information, derived from nature, but not limited to, hereditary units, metabolites, proteins, enzymes, prions, phenotypic expression and non-human cultures” (Ruiz Muller, 2015). In addition, this model proposes a shift of the disclosure of any information (including DSI) from a given resource, up to the point of its commercial use or patenting (when it happens) and following standardized royalties rates (Ruiz Muller, 2015). The rationale of such a model is that local case-to-case deals (as in the current Nagoya Protocol framework) are probably less fair, or at least less transparent than a global governance. Arguably, this model might be practically extremely complicated and would provide a strong discretionary power to the patent offices that might not follow the same (ecological, cultural, scientific, educational…) goals as other instruments like the Seed Treaty or the CBD. Also, it is unclear to which extent such a “bounded openness” regime might interact with other intellectual property regimes like farmer’s or breeder’s right, or when PGRFA bulks are used collectively in gene bank genomics approaches. Another “flavor” of such model has recently been proposed for marine genetic resources in areas beyond national jurisdiction, with default open access and an optional payable embargo period on data and samples (Vanagt et al., 2019). Concerning PGRFA, some stakeholders may find such models that consider natural information too reductionist and not embracing the socio-economical relationship that could exist between PGRFA and the population or group they may originate from.
- Another possible way of dealing with the increasing complexity of PGRFA governance is to try building an extensive “knowledge commons,” that can include all possible aspects of PGRFA (Winter, 2013), i.e., a global tax associated with any value-generating object or activity based on PGRFA (Halewood et al., 2018a; Halewood et al., 2018b). This again may require an adjustment of PGRFA definition to move away from the material-centric view and take into consideration the informational nature of the PGRFA. The main advantage here is the uncoupling of the access and use of the PGRFA and derived DSI. Taxing users, institutions, companies, or even countries will provide a possibly simpler system but also requires political consensus *via* new multilateral agreements.

### DSI as Common Heritage of Mankind?

The preamble of the Seed Treaty recognizes the PGRFA as a “common concern of all countries” (ITPGRFA, 2004). This precise formulation responded to the early days of multilateralism, where the Moon, the outer space, and the deep ocean were largely inaccessible and legally defined as common good of mankind. Despite the maybe quixotic goals that were initially aimed at, one can wonder to which extent any genetic resource and the information they hold could for example reasonably be managed under a protection similar to the cultural or natural heritages (UNESCO Convention Concerning the Protection of the World Cultural and Natural Heritage, 1972). However, it is clear that the actual inclusive governance of the genetic resources was designed in response to the earlier failures of multilateral approaches to protect biodiversity globally (Smouts, 2005; Blasiak et al., 2018). Preserving genetic resources is still a global priority, but this should not necessarily be taken as an argument to transform PGRFA as purely monetary commodity depleted of any ethical or sociological value. Proof is still to be made to which extent PGRFA and more globally genetic resource conservation actually benefited globally from the existing governance (Prathapan et al., 2018). This holds even more true when the commodity is a string of nucleotides freely accessible online originating from the other side of the globe in a changing environment. DSI as global “open access” common good would avoid the potential complications of any attempt to track and trace DSI at a global level (including possible mechanisms that might try maintaining compliance). Considering DSI as a common good of humankind would most probably raise major opposition related to the State’s sovereignties of each country on their genetic resources, as ruled in the CBD (Halewood, 2013). If considering genetic resources as a common heritage of mankind or more likely as a global public good, care should be taken to design a new inclusive form of governance that takes into consideration what exactly are these goods, how common they might be, and who exactly is the (hu)mankind.

## Actionable Recommendations

The DSI debates in various international fora have revealed pre-existing weakness in the design of the Seed Treaty, as well as possibly in other treaties dealing with genetic resources. This has at least two main consequences: firstly, there is a need to coordinate efforts to embrace scientific progress in the genetic resource field (Laird and Wynberg, 2018). A non-reductionist and pragmatic definition of PGRFA is urgently needed. The “new” PGRFA definition would ideally become a more inclusive concept that would better acknowledge farmer’s role in diversity creation and conservation (Bonneuil, 2019). Secondly, in light of what the various ongoing debates about genetic resources from many different sectors, care should be taken to understand the specificities of the PGRFA: modern breeding considers DSI-PGRFA largely in a “big data” manner, which might not be true—for example, for conservation of specific endangered species or public health. Interesting parallels can help improving the PGRFA framework, like other genetic resources originating from animals or bacteria (Dedeurwaerdere, 2013) or “classic” digital artifact governance (Stuermer et al., 2017). To allow a sustainable use of digital artifacts (DSI) originating from PGRFA (if totally uncoupled from the physical resource), some fundamental modifications of the digital infrastructure would be necessary: decentralization of the databases in modular structures and diversified funding are required to insure a resilient and fair system (Stuermer et al., 2017). Properly answering the challenges of dematerialization is a necessary condition to ensure that the ABS instruments stay relevant in the state of actual science and fulfill their objectives.

“Omics” has changed the precision and efficiency of breeding by an order of magnitude. Genomic selection and GWAS together with gene bank genomics approaches are becoming commonplace, even for some neglected and underused crops. For the Seed Treaty itself, DSI should be considered as a chance to embrace an extended multilateral approach by developing an updated and fair subscription system. However, such an approach should also carefully consider modalities of DSI generation, curation, storage, and dissemination. It will be essential to engage with various stakeholders to reduce disparities and encourage accessibility, transparency, and accountability (Bezuidenhout et al., 2017; Kaye et al., 2018). This might, in turn, reduce the “*breeding divide*” between low- or middle- and high-income countries. Large-scale deployment of genomics facilitated by a constant decrease of technology costs, democratization of open-source analytic pipelines, and capacity building may in the long term favor a major reshuffling of the way breeding is performed in various agroecosystems. Farmer-centric participatory breeding networks promote an integrative, bottom-up vision of plant breeding (Joly and Hervieu, 2003; Bonneuil and Thomas, 2009). However local-level PGRFA productions do not accommodate well with the Seed Treaty and the multilateral system (Halewood, 2013). Supporting wider use of genomic-based technologies for on-farm and community-based breeding may possibly allow emergence of new locally adapted diversity that is essential for crop improvement (Jarvis et al., 2008; Halewood et al., 2018b). A recent large-scale study by CGIAR showed the potential of crowdsource citizen-science programs to adapt plant varieties to site-specific conditions, a challenge for smallholder farmers suffering climate change (van Etten et al., 2019).

To conclude, the management of PGRFA and more generally of genetic resources appear to be at a turning point in its history. The digitization is now an underlying parameter of negotiations over the treaty’s reforms. It questions again the fragility and the limits of the Seed Treaty boundaries and capacities to trace efficiently DSI and their resources. This debate should be used as an opportunity to improve of the actual instrument in order to 1) think again what are the best possible ways to respond to the food security challenges, in other words, are any additional rule over DSI practicable? And 2) does the ABS framework, and the Seed Treaty in particular, focus on the wrong type of incentive? Alternative models of digital goods management are based on motivations to contribute to the commons pool without direct incentive from property rights. Reputation gain, learning effects or faster time to the market could be other alternative incentives to participate to the commons (Dedeurwaerdere, 2013; Stuermer et al., 2017). With the ongoing omics developments, if not correctly addressed, the “DSI issue” might threaten the stability of the Seed Treaty and the possibly the entire ABS framework. More work is needed to evaluate the relevance of the proposed models for each specific sector and the potential impact of digital “free-riding” on science, innovation, breeding, and ultimately food supply. Taking advantage of operational research methods, sustainability prediction of each particular model presented here is possible in a more quantitative way by applying robust decision-analytic tools used in other policy-making fields (Wohlfender-Bühler et al., 2016; Zheng et al., 2016). Whatever the most appropriate model of treaty-based international regulatory regime for PGRFA and their commons may be (by a reform of the existing instruments or by a new overarching treaty that would specifically fit to digital genetic resources), it appears already clearly that an increased degree of multilateralism and harmonization is necessary to align to the current practices and unravel the formidable potentials originating from digitization of PGRFA.

This review aimed at providing DSI users and providers a glimpse of the complexity of the various intertwined policies ruling PGRFA. Integration of digital information in the PGRFA policy framework will greatly depend how these DSI actors become aware of the potential impact of their daily practice on the global debate. In parallel, this may also encourage experts having access to digital infrastructures to try helping empowerment of smallholder farmer communities and local cooperative networks to “omics” techniques applied to breeding. This could be a most efficient, resilient, and decentralized model for a modern governance of PGRFA. This is essential, not only to save existing crop biodiversity, but also to create new genetic diversity that will strongly be needed to tackle some of the major global challenges humanity currently faces like climate change and food security.

## Author's Note

The views and opinions expressed in this article are those of the author and do not necessarily reflect the official policies or positions of the Federal Office for Agriculture (FOAG).

## Author Contributions

SA wrote this review.

## Funding

This work was supported by the Swiss National Science Foundation (#31003A_149389/1 ; #31003A_172977).

## Conflict of Interest Statement

The author declares that the research was conducted in the absence of any commercial or financial relationships that could be construed as a potential conflict of interest.
